# Development and Evaluation of a Novel and Rapid Detection Assay for *Botrytis cinerea* Based on Loop-Mediated Isothermal Amplification

**DOI:** 10.1371/journal.pone.0111094

**Published:** 2014-10-20

**Authors:** Ya-Bing Duan, Chang-Yan Ge, Xiao-Ke Zhang, Jian-Xin Wang, Ming-Guo Zhou

**Affiliations:** College of Plant Protection, Nanjing Agricultural University, Nanjing, Jiangsu Province, China; Naval Research Laboratory, United States of America

## Abstract

*Botrytis cinerea* is a devastating plant pathogen that causes grey mould disease. In this study, we developed a visual detection method of *B. cinerea* based on the *Bcos5* sequence using loop-mediated isothermal amplification (LAMP) with hydroxynaphthol blue dye (HNB). The LAMP reaction was optimal at 63°C for 45 min. When HNB was added prior to amplification, samples with *B. cinerea* DNA developed a characteristic sky blue color after the reaction but those without DNA or with DNA of other plant pathogenic fungi did not. Results of HNB staining method were reconfirmed when LAMP products were subjected to gel electrophoresis. The detection limit of this LAMP assay for *B. cinerea* was 10^−3 ^ng µL^−1^ of genomic DNA per reaction, which was 10-fold more sensitive than conventional PCR (10^−2 ^ng µL^−1^). Detection of the LAMP assay for inoculum of *B. cinerea* was possible in the inoculated tomato and strawberry petals. In the 191 diseased samples, 180 (94.2%) were confirmed as positive by LAMP, 172 (90.1%) positive by the tissue separation, while 147 (77.0%) positive by PCR. Because the LAMP assay performed well in aspects of sensitivity, specificity, repeatability, reliability, and visibility, it is suitable for rapid detection of *B. cinerea* in infected plant materials prior to storage and during transportation, such as cut flowers, fruits and vegetables.

## Introduction

*Botrytis cinerea* [teleomorph *Botryotinia fuckeliana*] is a necrotrophic, ascomycetous, phytopathogenic fungus with worldwide distribution. This fungus can infect at least 200 mostly dicotyledonous plant species including many agriculturally important and economically valuable crops. Grey mould caused by *B. cinerea* is a destructive disease and leads to serious losses in yield and quality on numerous crops, particularly fruits, vegetables, and ornamental flowers [1]. Thus, rapid detection of *B. cinerea* in early diagnosis of infected tissues and prior to storage of fruit or other commodities is important not only for maintaining yield and quality of agricultural products but also for controlling the pathogen’s spread.

The most common methods currently used for the detection of *B. cinerea* are based on conventional polymerase chain reaction (PCR) [2]–[7], plate-trapped antigen enzyme linked immunosorbent assay (PTA-ELISA) [8], and microfluidic chip assay (MCA) [9]. These methods are valuable tools for investigating latent infection and the early stages of disease. However, PCR has several intrinsic disadvantages, including the requirement of rapid thermal cycling, insufficient specificity, and rather low amplification efficiency [10], [11]. Both PTA-ELISA and MCA suffer from complex operation, consuming time, and high cost [8], [9]. Taking such disadvantages into account, a recent DNA amplification technique known as loop-mediated isothermal amplification (LAMP) was adapted for the detection of *B. cinerea*.

Loop-mediated isothermal amplification (LAMP), a novel technique, has been developed, which can amplify nucleic acids with high specificity, sensitivity and rapidity under isothermal conditions [12]. The method is easy to perform [13], [14], based on the principle of the reaction performed by a DNA polymerase with strand displacement activity and a set of two specially designed inner primers (FIP and BIP) and two outer primers (F3 and B3). LAMP products can be visualized with the naked eye by adding DNA-intercalating dyes such as ethidium bromide, SYBR Green I, propidium iodide, or Quant-iT PicoGreen; by adding metal-ion indicators such as hydroxynaphthol blue (HNB) [15], CuSO_4_ [16], or calcein [17], or by measuring the increase in turbidity derived from magnesium pyrophosphate formation (to infer increases in amplified DNA concentration). LAMP products can also be detected by real-time detection methods [18]. The simplicity of the LAMP method, which does not require a thermal cycler, makes it suitable for field testing.

Although the detection method of *B. cinerea* based on LAMP has been reported [19], the detection sensitivity was lower and we failed to detect *B. cinerea* in the field samples. In addition, reaction products were not visualized until gel electrophoresis with ethidium bromide staining. In the current study, we developed a LAMP assay with HNB for detection of *B. cinerea* based on the *Bcos5* gene (BC1G_07633) and demonstrated that the assay was simple, sensitive, specific and efficient. The new LAMP assay will provide important reference data for the detection of *B. cinerea* prior to storage of fruit or other commodities, or monitoring and controlling grey mould caused by *B. cinerea*.

## Materials and Methods

### Ethics statement

Our study does not involve human specimens or tissue samples, or vertebrate animals, embryos or tissues. In our study, *B. cinerea* isolates were collected from different private lands (indicated in table 1), and the field studies did not involve endangered or protected species. Chen Y. (email: 285920599@qq.com) should be contacted for future permissions.

**Table 1:** Plant samples used in this study, and numbers that were positive in the LAMP, PCR and tissue separation assays.

Hosts	Tissues	Geographical origin(private land)	Number ofsamples	Positivein LAMP	Positivein PCR	Positive intissue separation
Strawberry	Leaf	Lishui, Jiangsu	23	21	18	19
Strawberry	Fruit	Lishui, Jiangsu	56	55	43	53
Celery	Leaf	Yancheng, Jiangsu	14	13	13	13
Tomato	Leaf	Shouguang, Shandong	35	34	26	35
Tomato	Fruit	Shouguang, Shandong	23	21	19	18
Cucumber	Leaf	Yancheng, Jiangsu	11	9	6	8
Cucumber	Fruit	Yancheng, Jiangsu	29	27	22	26

### Fungal strains and reagents

*B. cinerea* isolates used in this work were collected from infected host leaves and fruits listed in Table 1. All tested *B. cinerea* isolates were obtained by the single-spore method. Other plant pathogens used in this study were maintained in the Laboratory of Plant Disease Control and Phytopharmacy, Nanjing Agricultural University, China, and are listed in Table 2.

**Table 2:** Species or populations of major plant pathogens used to evaluate the analytical specificity of the LAMP assay.

Species	Original host	Geographical origin (private land)	PCR	LAMP
*Botrytis cinerea*	Strawberry	Nanjing, Jiangsu	+	+
	Tomato	Nanjing, Jiangsu	+	+
*Sclerotinia sclerotiorum*	Rape	Jiangyan, Jiangsu	−	−
*Fusarium graminearum*	Wheat	Nantong, Jiangsu	−	−
*Rhizotonia cerealis*	Wheat	Taizhou, Jiangsu	−	−
*Rhizoctonia solani*	Rice	Jurong, Jiangsu	−	−
*Verticillium dahliae*	Cotton	Yangling, Shaanxi	−	−
*Alternaria alternata*	Tomato	Luoyang, Henan	−	−
*Colletotrichum gloeosporioides*	Pepper	Yancheng, Jiangsu	−	−
*Magnaporthe grisea*	Rice	Zhenjiang, Jiangsu	−	−

‘+’ and ‘−’ represent positive and negative results, respectively.

Bst DNA polymerase was purchased from NEB. Betaine and hydroxynaphthol blue (HNB) were purchased from Sigma, and MgCl_2_ and dNTPs were purchased from Takara. Double-distilled water (ddH_2_O) was used in all experiments. All other reagents were analytical grade.

### Culture conditions and DNA extraction

Fungi were cultured in potato dextrose broth (L^−1^, 200 g potato and 20 g dextrose mixed with distilled water). Mycelia of each isolate were harvested by filtration and frozen at −20°C. Mycelia DNA was extracted using the Plant Genomic DNA Kit (Tiangen, Beijing) according to the manufacturer’s instructions and was quantified by spectrophotometry, and extracted DNA was stored at −20°C.

### Primer design

**Figure 1:**
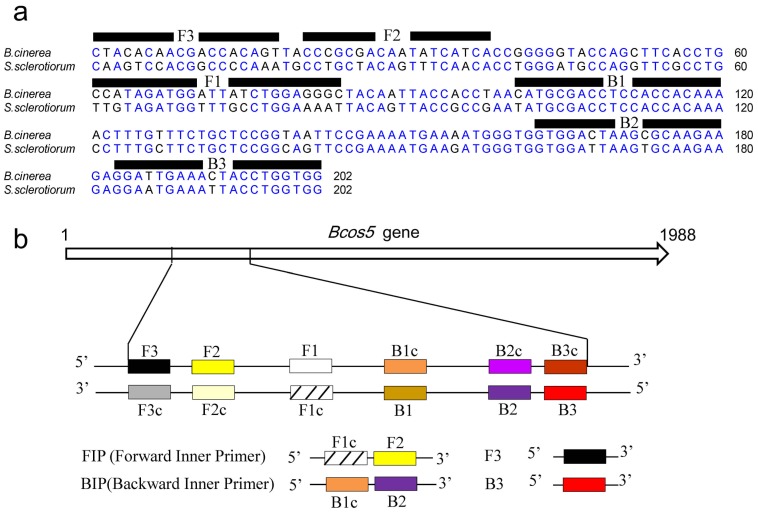
Design of LAMP primers for detection of *B. cinerea*. (a) Nucleotide sequence alignment of the target region *Bcos5* in *B. cinerea* and *Ssos5* in *S. sclerotiorum*. The sequences used for LAMP primers are indicated by bold lines. (b) Schematic representation of the LAMP primers used in this study. Construction of the inner primers FIP and BIP are shown. F1c and B1c are complementary to F1 and B1, respectively.

PCR primers (Table 3) were designed using Primer Premier 5.0 (Premier, Canada) and Oligo 6.0 (MBI, Cascade, CO) software. Four specific LAMP primers were designed based on the *B. cinerea Bcos5* gene (BC1G_07633). A BLASTN search indicated that the sequence of *Bcos5* had 82% similarity with that of *Ssos5* (SS1G_14143) of *S. sclerotiorum*. Then, we obtained the *Bcos5* and *Ssos5* sequences in the genome databases for *B. cinerea* and *S. sclerotiorum* (http://www.broadinstitute.org/), respectively. In this way, specific primers based on the *Bcos5* sequence alignment were designed for LAMP detection of *B. cinerea* (Fig. 1a) using the Primer explorer V4 software program (http://primerexplorer.jp/e/). The structure of the LAMP primers and their complementarity to target DNA used in this study are shown in Fig. 1b. A forward inner primer (FIP) consisted of F1c and F2, and a backward inner primer (BIP) consisted of B1c and B2. The outer primers F3 and B3 were required for initiation of the LAMP reaction. Primer pair P1/P2 was used for conventional PCR of the *B. cinerea Bcos5* gene. Information regarding the primer names and sequences is provided in Table 3.

**Table 3:** Information on the primers used in this study.

Primer name	Sequence (5′-3′)	Length (bp)
F3	CTACACAACGACCACAGT	18
B3	CCACCAGGTAGTTTCAATCC	20
FIP	GCCCTCCAGATAATCCATCTATGG-CCCGCGACAATATCATCA	42
BIP	CATGCGACCTCCACCACAAA-TTCTTGCGCTTAGTCCAC	38
P1	GATACCCCTCAACAAAAGCCT	21
P2	CCAGGTTGTCTTCCTACTTGC	21

### Optimization of LAMP reaction

The LAMP reaction was performed in a total volume of 25 µL. For optimization of reagents, a range of concentrations of Bst DNA polymerase large fragments (0.16–0.64 U µL^−1^), dNTPs (0.2–2 mM), Mg^2+^ (2–8 mM), primers (0.2–2 µM), betaine (0.8–1.6 M), and HNB (100–200 µM) were evaluated. The reaction was performed in 0.2-mL micro centrifuge tubes that were placed in a water bath for 1.5 h at 65°C and then heated for 10 min at 80°C to terminate the reaction. Template DNA was replaced by ddH_2_O as negative control. Each product was analyzed by 3.0% agarose gel electrophoresis stained with ethidium bromide and photographed under a UV transilluminator. In addition, the amplification product could also be visually inspected according to the color change from violet to sky blue, while the negative control remained violet. The tubes were observed by naked eyes and photographed under the natural light.

### Optimization of LAMP reaction conditions

The LAMP reaction mixtures were incubated for 45 min at 61, 62, 63, 64, or 65°C to determine the optimal reaction temperature. Then, the LAMP was performed at the optimal reaction temperature (63°C, see Results) for 15, 30, 45, 60, and 90 min to determine the optimal reaction time (45 min, see Results). The reactions were terminated by heat inactivation at 80°C for 10 min. The assays were assessed based on HNB-visualized color change and then on gel electrophoresis as described in the previous section. The intensity of DNA on gel electrophoresis was analyzed by the software Quantity One (BioRad).

### Sequencing of LAMP products

After electrophoresis, 202-bp DNA bands obtained from the positive LAMP reaction on gel were extracted using a Gel Extraction Kit (Omega, USA), and were amplified by PCR using the primers F3 and B3. PCR reaction mixtures contained 10 µM F3 and B3 (2 µL each primer), 2.5mM dNTPs (2 µL), 10× PCR buffer (Mg^2+^ Free, 2.5 µL), 25 mM MgCl_2_ (1.5 µL), 5 UµL^−1^ rTaq (0.125 µL), template DNA (1 µL), and ddH_2_O (13.875 µL). PCR reactions were performed as follows: 94°C for 2 min, and then 35 cycles of denaturation at 94°C for 30 s, annealing at 54°C for 30 s, extension at 72°C for 30 s, with a final extension at 72°C for 10 min. The 202-bp product was extracted after 1% agarose gel electrophoresis, cloned into *pEASY*-T1 Cloning Vector (Transgen, Beijing), and then transformed into competent TOP10 cells. The recombinant plasmid *pEASY*-T1-N202 was extracted from positive clones and sequenced by Sangon (China).

### Restriction enzyme digestion analysis of LAMP products

LAMP products were digested in a 20 µL reaction system containing Kpn I (1 µL) (Takara), 10×L Buffer (2 µL), LAMP products (8 µL), and ddH_2_O (9 µL), incubated at 37°C overnight, after which the DNA band were analyzed on 3% agarose gel electrophoresis stained with ethidium bromide and photographed as above.

### LAMP specificity test

LAMP specificity was verified by performing the assay of DNA of *B. cinerea* and other important plant-pathogenic fungi in agricultural production, including *Sclerotinia sclerotiorum* [20], *Fusarium graminearum* [21], *Rhizoctonia cerealis* [22], *Rhizoctonia solani* [23], *Verticillium dahliae* [24], *Alternaria alternata* [25], *Colletotrichum gloesporioides* [26], and *Magnaporthe grisea* [27]. The LAMP assay was done as described earlier. As before, the assays were assessed based on HNB-visualized color change and gel electrophoresis. Each fungal sample had three replications, and the experiment was performed three times.

### LAMP repeatability test

To evaluate the accuracy of LAMP detection of *B. cinerea*, *B. cinerea* isolates from diseased celery leaf (n = 14), tomato (n = 36) and strawberry (n = 58) fruits were tested and a DEPC-treated water as negative control under the same condition.

### Sensitivity comparisons of LAMP and PCR

Various concentrations of total DNA were used as templates in LAMP and PCR. Template DNA was prepared as described earlier and was 10-fold serially diluted from 1 ng µL^−1^ to 10^−7^ ng µL^−1^ prior to use as the template in LAMP and PCR. Based on the DNA sequence of *Bcos5*, the primers P1/P2 were designed for PCR detection. PCR reactions were performed as follows: 94°C for 2 min, and then 35 cycles of denaturation at 94°C for 30 s, annealing at 57°C for 30 s, extension at 72°C for 30 s, with a final extension at 72°C for 10 min. The process and conditions of the sensitivity test were similar to the specificity test for LAMP described as above. When the reactions were completed, the products were analyzed on 3% agarose gel. The test was performed three times.

### Application of LAMP on petal inoculum of *B. cinerea*

Tomato and strawberry petals from healthy glasshouse-grown plants were excised and washed with sterile-distilled water for three times, blotted with sterile filter paper to remove excess water, and allowed to dry. The petals were sprayed with water and spore suspension (1.0×10^4^ mL^−1^) and placed at 25°C with a 12 h photoperiod and 80% relative humidity. After 24 h, the inoculated petals were ground in 2 mL centrifugal tubes using the tissue lyser (MM400, Retsh). DNA was extracted using the method as described above. There were twenty replicates per host plant, and the experiment was repeated twice.

### Evaluation of LAMP on diagnosis of grey mould by *B. cinerea*

To evaluate the LAMP assay for detection of grey mould caused by *B. cinerea*, 191 diseased tissues and residues collected from different areas of China in 2013 (Table 1) were tested by the LAMP assay and PCR, as described above. Isolation of *B. cinerea* from these samples was also performed using a tissue separation method [28].

## Results

### Optimization of LAMP reaction

**Figure 2:**
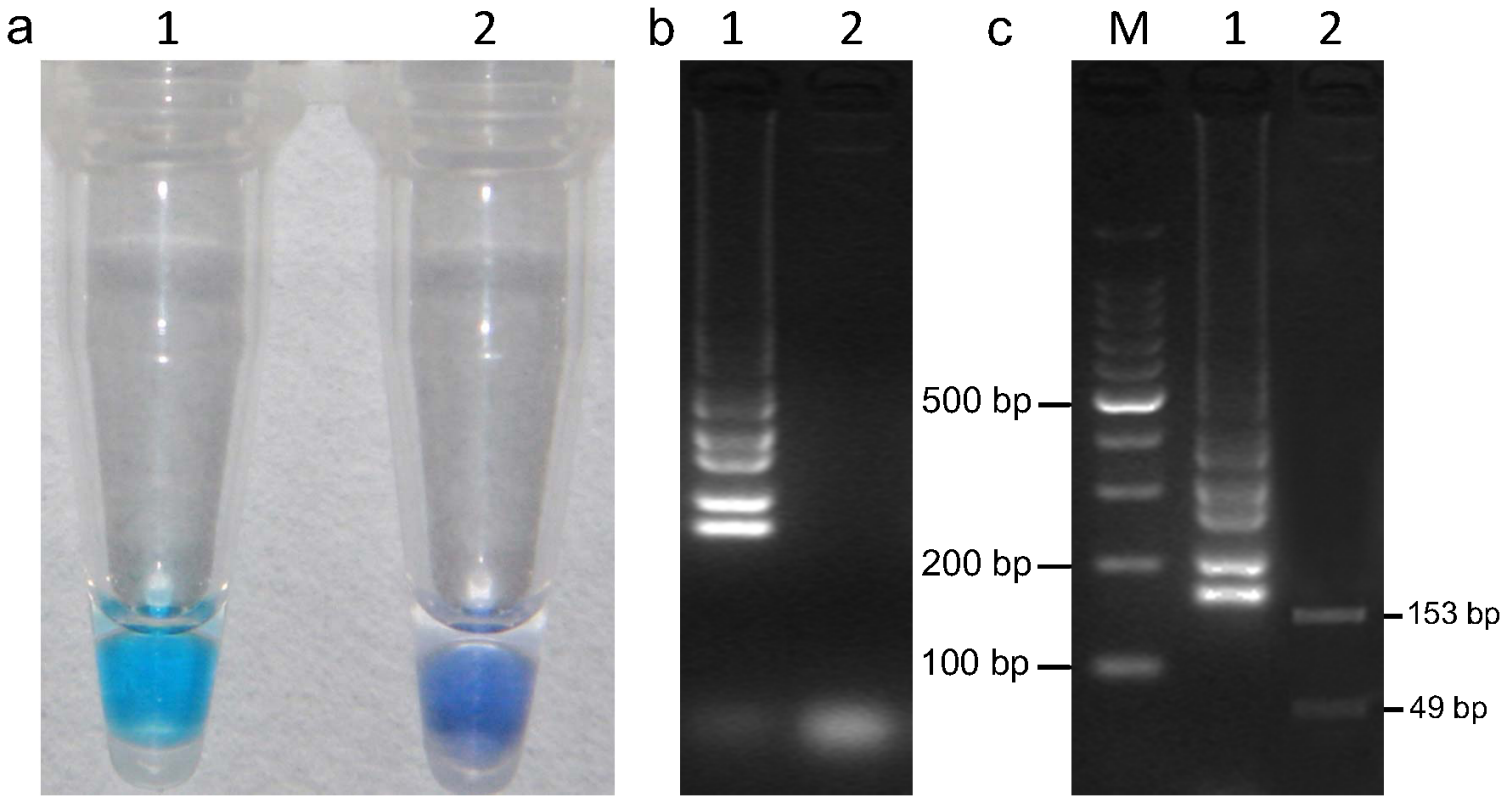
LAMP detection of the *Bcos5* gene in *B. cinerea* and digestion of positive LAMP products. (a) LAMP for detection of *B. cinerea* using HNB as a visual indicator. The reaction becomes sky blue if the *Bcos5* gene is present but remains violet if the gene is absent; (b) Agarose gel electrophoresis of LAMP products. The positive reaction is manifested as a ladder-like pattern on the 3.0% agarose gel. In (a) and (b), the positive reaction (with target DNA) is labeled “1″, and the negative reaction (without target DNA) is labeled “2″; (c), LAMP products were digested with Kpn I, and two fragments (153 bp, 49 bp) were observed by 3.0% agarose gel. M = 100-bp ladder, 1, LAMP products without digestion; 2, LAMP products digested by Kpn I.

When the LAMP assay was performed with *B. cinerea* DNA as the template, the best results were obtained in a 25 µL volume containing 8 U of Bst DNA polymerase, 2.5 µL 10×ThermoPol, 3 mM MgCl_2_, 1 mM dNTPs, 1.2 µM each of FIP and BIP, 0.2 µM each of F3 and B3, 0.64 M betaine, 150 µM HNB, and 1 µL of target DNA. The reactions were performed in a 0.2-mL centrifuge tube in water bath for temperature control. When the tubes were examined before gel electrophoresis, a positive LAMP reaction was indicated by a sky blue color; while the color remained violet for negative reaction (Fig. 2a). After the tubes were visually assessed by color change, the samples were analyzed by agarose gel electrophoresis. As expected, the typical ladder-like pattern on 3.0% agarose gel electrophoresis was observed in a positive sample, but not in the negative control (Fig. 2b). The results showed that the primers were effective, and the same result was obtained with HNB visualization and gel electrophoresis.

### Optimization of LAMP reaction conditions

**Figure 3:**
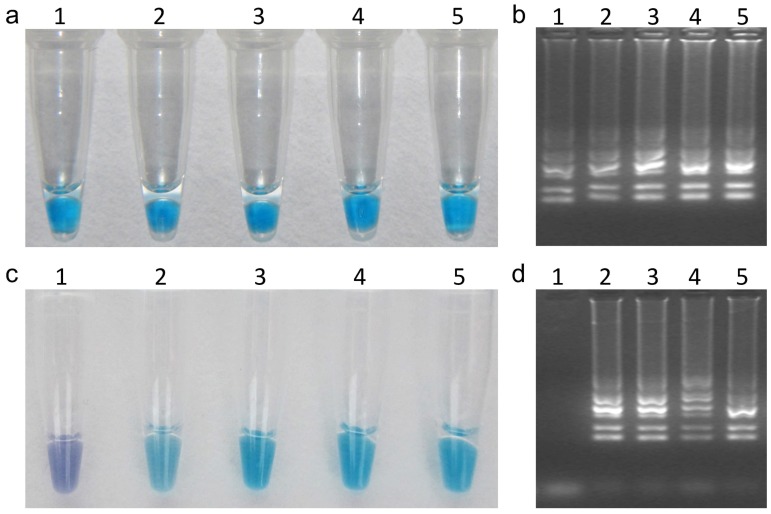
Optimization of LAMP reaction temperature (a, b) and reaction time (c, d). Assessment was based on HNB visualization of color change in (a) and (c) and on gel electrophoresis in (b) and (d). In (a) and (b), 1 = 61°C, 2 = 62°C, 3 = 63°C, 4 = 64°C, and 5 = 65°C. In (c) and (d), 1 = 15 min, 2 = 30 min, 3 = 45 min, 4 = 60 min, and 5 = 90 min.

According to the reaction reagents optimized as indicated in the previous section, LAMP was performed using *B. cinerea* DNA as template to determine the optimal reaction temperature and time. Color change at different temperatures was consistent (Fig. 3a); however, the intensity of DNA at 63°C was the strongest among all of the test temperatures (Fig. 3b). When LAMP was performed at 63°C with a range of test time, positive results were obtained with time from 30 to 90 min whether assessment was based on HNB-visualization (Fig. 3c) or gel electrophoresis (Fig. 3d), but the color at 30 min was pale (Fig. 3c). Therefore, the ideal reaction condition of the LAMP for *Bcos5* gene was 63°C for 45 min (Fig. 3).

### Sequencing and restriction endonuclease digestion of LAMP products

As shown in Figure 2b, LAMP products of *B. cinerea* showed a ladder-like pattern on agarose gel electrophoresis. When just ddH_2_O without template, was used, no amplification was seen (Figure 2b). In addition, LAMP products could be observed by naked eyes under the natural light. The negative control reaction showed violet, but the color became sky blue in positive amplification (Figure 2a). To further confirm that LAMP products were the expected target sequence, sequence analysis was determined. Sequencing results of the recombinant plasmid *pEASY*-T1-N202 indicated that the 202 bp target sequence was 100% homologous to the sequence of *Bcos5* gene used for the primers design (data not shown). After digestion of LAMP products with the Kpn I, the 49 and 153 bp fragments were observed (Figure 2c), and were in good accordance with those predicted theoretically from the expected structures. The results of the sequence and the digestion confirmed that the products were amplified specifically from the *Bcos5* target region.

### Specificity of the LAMP assay

**Figure 4:**
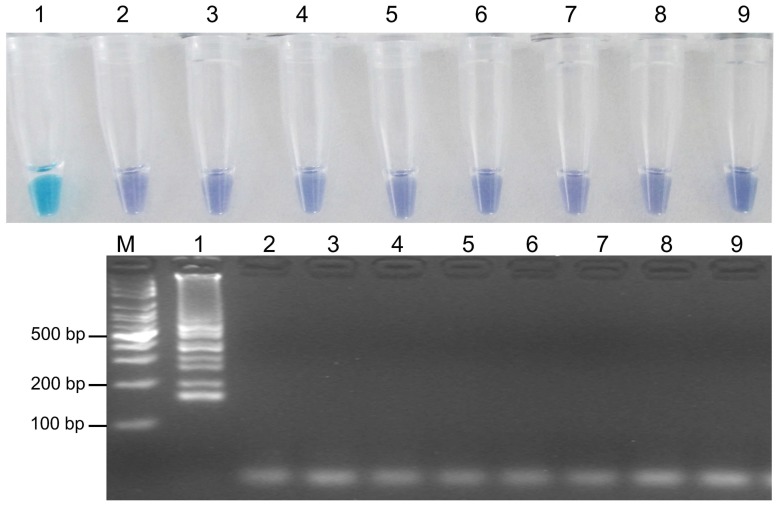
Specificity of LAMP detection of *B. cinerea*. Assessment was based on (a) HNB visualization of color change or (b) agarose gel electrophoresis analysis of the LAMP products. M indicates a 100-bp ladder; 1, *B. cinerea*; 2, *S. sclerotiorum*; 3, *F. graminearum*; 4, *R. cerealis*; 5, *R. solani*; 6, *V. dahliae*; 7, *A. alternata*; 8, *C. gloesporioides*; 9, *M. grisea*.

The LAMP assay was positive only for *B. cinerea*, i.e., no positive DNA products were observed when other fungi (*S. sclerotiorum*, *F. graminearum*, *R. cerealis*, *R. solani*, *V. dahlia*, *A. alternata*, *C. gloesporioides*, and *M. grisea*) were used as templates. This was confirmed by both HNB-visualization (Fig. 4a) and gel electrophoresis (Fig. 4b).

### Repeatability of the LAMP assay

The robustness and repeatability of the LAMP method were tested with 108 known *B. cinerea* isolates from different hosts. All the isolates were positive by the LAMP assay described above (data not shown). The results indicated that the LAMP assay developed in this study was repeatable and stable.

### Sensitivity of the LAMP and PCR assays

**Figure 5:**
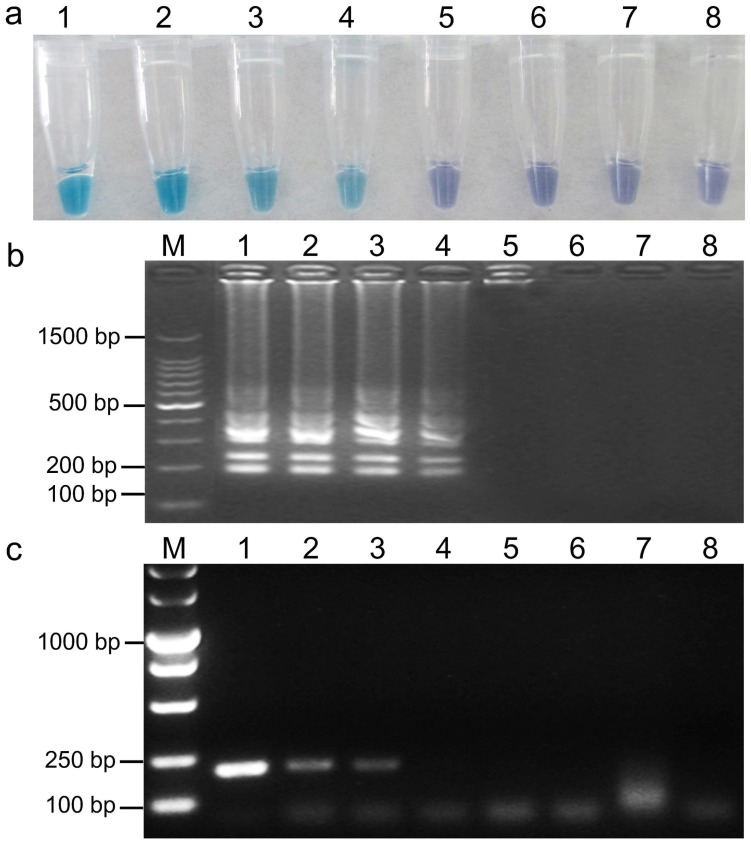
Sensitivity of LAMP vs. conventional PCR for detection of *B. cinerea* genomic DNA. Detection by (a) LAMP and HNB visualization, (b) LAMP and gel electrophoresis, and (c) conventional PCR. Concentrations of template DNA (ng µL^−1^) per reaction in (a), (b), and (c) were: 1 = 10°, 2 = 10^−1^, 3 = 10^−2^, 4 = 10^−3^, 5 = 10^−4^, 6 = 10^−5^, 7 = 10^−6^, and 8 = 10^−7^. In (b) and (c), M indicates 100-bp, and DL 5000 ladder, respectively.

To determine the detection limit, PCR and LAMP were performed using 10-fold serial dilutions of *B. cinerea* genomic DNA. PCR products were detected by agarose gel electrophoresis, and a 233 bp specific band could be seen clearly (Fig. 5c). LAMP products were detected both by visual inspection and by agarose gel electrophoresis (Fig. 5a, 5b). As shown in Fig. 5, LAMP was successfully amplified when the template was diluted to 10^−3 ^ng µL^−1^ (Fig. 5a, 5b), whereas PCR could only be amplified when diluted to 10^−2^ ng µL^−1^ (Fig. 5c). Thus, LAMP was 10-fold more sensitive than PCR.

### Application of LAMP on petal inoculum of tomato and strawberry

The aim of these experiments was to determine whether the inoculated petals could be detected or not using LAMP in the presence of a high background of tomato and strawberry DNA. In the experiments, DNA purified from uninoculated petals and petals inoculated by *B. cinerea* spore suspension were tested using the LAMP assay. All the assays of inoculated petals were positive whether assessment was based on HNB-visualization or gel electrophoresis, whereas those of the uninoculated petals were negative. This showed that the LAMP assay could successfully detect the infection of *B. cinerea* on tomato and strawberry petals.

### Evaluation of LAMP on diagnosis of grey mould by *B. cinerea*

To demonstrate the applicability of the LAMP assay in field samples, 191 plant materials infested with *B. cinerea* were tested by LAMP, PCR, and tissue separation. The positive-sample ratios were 147/191 (77.0%) by conventional PCR, 180/191 (94.2%) by the LAMP assay, and 172/191 (90.1%) by the tissue separation method (Table 1). Additionally, all PCR positive samples could be detected by LAMP. This result suggested that the LAMP method was more feasible than PCR when detecting *B. cinerea* from diseased samples for its higher sensitivity. Therefore, the LAMP assay reported here might be used for visual detection of *B. cinerea* in plants and production fields.

## Discussion

Grey mould (*B. cinerea*), causing dieback and decay of most plant parts, is a major threat to the global agricultural production [29]. Rapid and accurate detection of *B. cinerea* is vital to prevent the introduction and spread of *B. cinerea* into non-endemic areas, and to control and minimize damage to the agricultural production. Although rapid detection of *B. cinerea* by LAMP has been reported [19], we failed to detect *B. cinerea* in the field samples using this method. In addition, reaction products of LAMP were not visualized by naked eyes, but by staining with ethidium bromide following gel electrophoresis. Taking such disadvantages into account, we first reported a visual detection of *B. cinerea* based on the *Bcos5* gene using LAMP with HNB. The diagnosis of grey mould in agricultural production using LAMP was also developed. Compared with conventional PCR, the LAMP assay reported here is more advantageous owing to its simpler operation, more rapid reaction, and easier detection. In this study, the optimal temperature and time of LAMP for detection of *B. cinerea* were determined to be 63°C, and 45 min, respectively. Because LAMP is conducted at one temperature, no time is wasted as a result of changes in temperature, as is the case with thermal cycling with PCR. Moreover, LAMP requires only a regular laboratory bath or heat block that can provide a constant temperature of 63°C. Another very important advantage of LAMP is that the amplified products can be visually detected by adding the dye HNB, i.e., electrophoresis is not required. Because the LAMP assay is simple and relatively easy to perform, it should be useful even for those laboratories and research institutes that are unfamiliar with PCR or other methods of molecular analysis.

It is reported that the LAMP reaction might be facilitated by the addition of loop-forward and loop-backward primers [30]. In the present study, suitable loop-forward and loop-backward primers were not identified, and only four primers (F3, B3, FIP, and BIP) were used to develop the LAMP assay for detection of *B. cinerea*. To confirm the efficiency and specificity of the four primers, we used DNA extracted from *B. cinerea* and from other important plant-pathogenic fungi as templates for LAMP assay. The LAMP assay could correctly distinguish *B. cinerea* from other pathogens, i.e., the LAMP assay and the primers designed here were specific for *B. cinerea*. Restriction enzyme and sequence analyses also validated its specificity.

As the LAMP reaction progresses, pyrophosphate ions are produced and bind with Mg^2+^ ions to form a white precipitate of magnesium pyrophosphate. Therefore, the results of the LAMP can be judged by naked eyes. This characteristic feature of the LAMP reaction requires staining with fluorescent intercalating dyes. SYBR green [31] and Picogreen [32], added after the reaction, have been developed to allow visual discrimination of positive samples. However, use of these intercalating dyes increases the rates of contamination because the tubes are open. To avoid such contamination, a visualization indictor (HNB) prior to amplification was used in the LAMP assay in this study. HNB is a colorimetric indicator of calcium and alkaline earth metal ions. In a LAMP reaction mixture, dNTPs can influence the color of HNB by the chelating with the Mg^2+^ ions. In the presence of HNB, the color gradually changes from violet to sky blue as the dNTPs decrease during amplification [15]. In this study, 150 µM HNB could successfully distinguish positive and negative samples. Compared with other methods used to visually detect endpoints, such as those based on the visualization of turbidity [33], the addition of DNA intercalating dyes [31], [32], [34], [35], or the use of calcein [17] and CuSO_4_ [16], the use of HNB is simpler [15], [28], [36]. HNB can be added before incubation so that the reaction tubes need not be opened, which reduces the risk of cross-contamination. The positive and negative reactions obtained with LAMP and HNB were confirmed when the LAMP products were subjected to gel electrophoresis to analysis.

108 known *B. cinerea* isolates from the different hosts were used to determine the robustness and repeatability of the LAMP method established in the current study. All the isolates were positive with LAMP, and amplicons were confirmed through 3% gel electrophoresis (data not shown). This indicated that the LAMP assay established in this study had good repeatability and stability.

The limit of detection of *B. cinerea* DNA using the LAMP method was 10^−3 ^ng µL^−1^. This detection limit was lower (i.e., the sensitivity is greater) than previously reported LAMP methods used to detect *Phytophthora sojae* and *Phytophthora* spp. [11], [28], [29], [37]. Compared with conventional PCR, the LAMP method with HNB dye to detect *B. cinerea* had significantly higher sensitivity levels. This result was concordant with previous reports of the LAMP method for detecting some plant pathogens [19], [28], [37], [38].

The risk of some plant diseases has been related to the petal infestation percentage at early bloom [39]. The detection methods for risk assessment of rape sclerotinia rot by *S. sclerotiorum* were developed based on infected petals [40]–[42]. Up to now, the risk assessment system of petals infected by *B. cinerea* has not been investigated. In this study, the LAMP method detecting *B. cinerea* on tomato and strawberry inoculated petals was successfully established. This suggested that the detection method was reliable when petals were infected by *B. cinerea* spores.

In the field trial, we collected 191 diseased tissues and residues from different host plants. All samples were tested by LAMP, PCR, and tissue separation method for comparison (Table 1). Compared with other methods, the newly developed LAMP significantly improved the detection efficiency. Thus, the LAMP assay developed in this study can be used for rapid and early diagnosis of *B. cinerea* in plants and production fields.

In summary, we have established a LAMP assay combined with HNB and demonstrated that it is more sensitive, specific, and practical for detection of *B. cinerea* than previous methods. Therefore, the new LAMP assay will be potentially useful for monitoring and controlling the occurrence of *B. cinerea* in agricultural production.
